# R. E. Caruthers

**Published:** 1885-03

**Authors:** Horace F. Ivins

**Affiliations:** Secretary


### R. E. CARUTHERS.

EDITOR "HOMŒOPATHIC PHYSICIAN," Philadelphia.

DEAR DOCTOR:—At the last regular meeting of the Society, held January 8th, the following resolutions were unanimously adopted:

"WHEREAS, R. E. Caruthers, M. D., of Allegheny, a valued member of the homœopathic medical profession, has been removed by death from his field of labor; and

"WHEREAS, We, the members of the Homœopathic Medical Society, of the County of Philadelphia, appreciating the great services rendered by Dr. Caruthers as Corresponding Secretary of the 'Homœopathic Medical Society of the State of Pennsylvania,' and recognizing the loss which Homœopathy in our State has sustained in his death, do hereby

"*Resolve,* That we extend our sympathy to the Homœopathic Medical Society of Allegheny County in their loss, and to his family in their bereavement.

"*Resolved,* That the Secretary be instructed to send a copy of these resolutions to the Allegheny County Society and to the family of the deceased, and to the homœopathic journals throughout the country.

"Committee signed,

CLARENCE BARTLETT, M. D.

J. C. GUERNSEY, M. D.

H. NOAH MARTIN, M. D."

Very respectfully,

HORACE F. IVINS, M. D.,
*Secretary.*

